# An efficient single shot detector with weight-based feature fusion for small object detection

**DOI:** 10.1038/s41598-023-36972-x

**Published:** 2023-06-19

**Authors:** Ming Li, Dechang Pi, Shuo Qin

**Affiliations:** 1 School of Computer Science and Technology, Nanjing University of Aeronautics and Astronautics, Nanjing, 211100 China; 2 Beijing Institute of Environmental Features, Beijing, 100854 China

**Keywords:** Computational science, Computer science

## Abstract

Object detection has been widely applied in various fields with the rapid development of deep learning in recent years. However, detecting small objects is still a challenging task because of the limited information in features and the complex background. To further enhance the detection accuracy of small objects, this paper proposes an efficient single-shot detector with weight-based feature fusion (WFFA-SSD). First, a weight-based feature fusion block is designed to adaptively fuse information from several multi-scale feature maps. The feature fusion block can exploit contextual information for feature maps with large resolutions. Then, a context attention block is applied to reinforce the local region in the feature maps. Moreover, a pyramids aggregation block is applied to combine the two feature pyramids to classify and locate target objects. The experimental results demonstrate that the proposed WFFA-SSD achieves higher mean Average Precision (mAP) under the premise of ensuring real-time performance. WFFA-SSD increases the mAP of the car by 4.12% on the test set of the CARPK.

## Introduction

In the last decades, computer vision is under extensive research in both academic and industrial applications, such as transportation surveillance, smart city, and sports analysis^1^. Object detection is the foundation of various other computer vision tasks including object tracking, action recognition, and image captioning. Object detection consists of two sub-problems, the location of the bounding boxes of objects and the classification of the object in each box. The previous traditional object detectors are based on the sliding-window paradigm and hand-crafted features^2–4^. However, traditional object detectors suffer from huge computational resources, redundant anchor boxes, and poor robustness.

In recent years, the effectiveness of convolutional neural networks (CNNs)^5^ has been verified in various fields of computer vision, CNN-based object detectors can be roughly divided into two-stage detectors (R-CNN^6^ and its variants^7,8^) and one-stage detectors (SSD^9^ and YOLO^10^). The two-stage detectors firstly identifying the potential regions may contain objects, and then classify the regions one by one. In most scenarios, two-stage detectors achieve excellent accuracy with slow detection speed. Conversely, one-stage detectors provide slightly poor detection accuracy with fast speed through merging the identification of regions and classification into one step. To be specific, one-stage detectors aim at mapping image pixels to coordinate of bounding boxes directly. Although the detection speed can be significantly enhanced, the detection accuracy in several scenes is insufficient.

Detecting the tiny object in images is highly challenging because of the ambiguous boundaries, complex backgrounds, and changing illumination. To address this problem, this paper proposes an efficient WFFA-SSD with a weighted-based feature fusion block and context attention block based on the original single-shot detector. In a weight-based feature fusion block, several multi-scale feature maps are fused with self-adaptive weight. Meanwhile, the context attention block is employed to reinforce the local feature in the feature maps. Therefore, two feature pyramids are fused to make predictions. The comprehensive experiments are conducted on the CARPK and PASCAL VOC datasets, and the experimental results demonstrate that the proposed WFFA-SSD outperforms the compared state-of-the-art one-stage object detectors. The contributions in this paper are summarized as follows.

- A weight-based feature fusion block is designed to adaptively fuse information of several multi-scale feature maps. Then, the feature maps with large resolutions can obtain more contextual information.
- A context attention block is applied to reinforce the local region in the feature maps.
- A pyramids aggregation block is employed to combine the two feature pyramids to classify and locate target objects.
- The extensive experiments on CARPK and PASCAL VOC dataset demonstrate that the proposed WFFA-SSD is superior to the other state-of-the-art one-stage detectors consistently.

The rest of this paper is organized as follows. In “Related work” section, the related works of object detectors, multi-scale feature fusion, and attention mechanism are introduced. “Proposed method” section elaborates the structure of the proposed WFFA-SSD and the the components. “Experimental results and analysis” section conducts the experiments and analyzes the experimental results. “Conclusions and future work” section presents the conclusion and the future work.

## Related work

In this section, a comprehensive review of the related work is provided. Firstly, the deep learning-based object detector is described. Then, multi-scale feature fusion is introduced. Moreover, the attention mechanisms in object detectors are discussed.

### Object detectors

Based on the excellent achievement of deep CNN in the field of computer vision, various deep learning-based object detectors perform superior performance when compared to conventional approaches. In R-CNN^6^, the two-stage scheme with the region proposals is designed. The region proposals are mainly employed to filter the potential regions with a high probability to involve objects. Then, the regions are provided to CNN for further prediction. As above procedure, the hand-crafted approaches are replaced by the two-stage deep learning-based object detectors with high accuracy and speed. Based on R-CNN, Fast R-CNN^7^ is introduced with a multi-task loss to train the classification and bounding box regression simultaneously. Then, Faster R-CNN^8^ is designed with a learning-based region proposal network (RPN) to further alleviate the computational cost of the proposals generation procedure. However, the detection speed of the two-stage detectors is insufficient in several scenes.

Compared with the two-stage object detectors, the one-stage object detectors abandon the proposals extraction to accelerate inference. YOLO^10^ is the first one-stage detector that performs classification and box regression directly on the pre-defined image grid. Unfortunately, the drop in region proposals results in low accuracy when the speed is accelerated. The improved versions of YOLO^11,12^ further improve the detection accuracy with high detection speed. In WOG-YOLO^13^, the whale optimization algorithm is employed to optimize the hyper-parameters of YOLOv5. Another popular branch of a one-stage detector is SSD^9^, which predicts with multi-reference and multi-scale feature maps. In RetinaNet^14^, focal loss is proposed to address the imbalance between positive and negative categories. Then, the RetinaNet achieves a trade-off between accuracy and detection speed. In EfficientDet^15^, a weighted bi-directional feature pyramid network (BiFPN) and a composite scale expansion method are designed. The accuracy of object detection is further improved. However, the detection accuracy of one-stage detectors especially on the small object is slightly poor when compared to the two-stage detectors.

As above, it is desirable to design a one-stage object detector with fast detection speed for small objects. Because of the distinct features of small objects in images, it is important to make full use of the information in the feature maps to further enhance the performance of object detectors.

### Multi-scale feature fusion

In most of the object detection tasks, the low-level feature maps with high resolution contain more location and detail information. However, the low-level feature maps lack semantic information. The high-level feature maps have more semantic information based on the convolution operator. The local information on high-level feature maps is poor. Therefore, multi-scale feature fusion is efficient to improve the performance of object detectors. The multi-scale feature fusion consists of the early fusion and late fusion strategy. In the early fusion strategy, the features maps are fused first, and then the fused features are employed to make predictions. For example, the addition operation in ResNet^16^ and the concatenation operation in U-Net^17^ are typical early fusion strategies. In the late feature fusion strategy, the detection results of several layers are combined to further enhance the performance of detectors. In the previous literature, several approaches independently make predictions based on the multi-scale features before fusion, and then the results of prediction are processed comprehensively, such as SSD^9^, multi-scale CNN^18^, etc. The other line applies the feature pyramid networks for reference and predict fusing the feature, such as YOLOv3^12^, FSSD^19^, etc.

The feature fusion strategy has been widely applied in object detection. To further refine the features extracted from VGG-Net, a discrimination correlation analysis (DCA)-based feature fusion strategy^20^ is proposed to enhance the performance of detectors with small computational cost. In FSSD^19^, a feature fusion module is designed to generate a novel feature map and constructed the feature pyramid. In the feature fusion module, multi-scale feature maps are combined through concatenation operators. Based on FSSD, the FS-SSD with an extra scaling branch and the spatial context analysis are proposed. In the scaling branch, the de-convolution module with an average pooling operation is employed to form a feature pyramid. Then, two feature pyramids are applied to make predictions together. In NLFFTNet^21^, the attention of different positions is calculated to capture the long-distance dependency, and the dynamic data augment method is employed to balance different classes. In MFABA^22^, a multi-scale feature aggregation module is applied to integrate adjacent hierarchical features and the multi-scale feature maps are combined to balance semantic feature information. In CSFF^23^, the feature pyramid network is employed to obtain multi-scale feature maps, and the CSFF is applied to obtain discriminative multi-level feature representations. In FPS-Net^24^, the multiple channel images are grouped into different modalities to obtain modality-specific features, and then map the features into a high-dimensional feature space for pixel-level fusion and learning.

In the Libra R-CNN^25^, the multi-scale feature maps are combined to obtain more balanced semantic feature information. The comparison of Faster R-CNN and RetinaNet demonstrates that the detection performance on the MS COCO dataset is significantly improved. In the neural architecture search feature pyramid network (NAS-FPN)^26^, a neural architecture search algorithm is employed to customize a feature pyramid network that merges features across a range. NAS-FPN produced significant improvements in many object detection networks. Besides, An adaptive spatial feature fusion (ASFF)^27^ strategy was proposed to combine multi-scale feature maps through learning weight parameters adaptively. The experimental results indicate that ASFF outperforms concatenation and element-wise addition. Through analyzing the weakness of only using deep learning methods, a new fusion logic^28^ which combines the advantages of knowledge used by a traditional method is proposed to further improve the performance of object detectors. To accelerate the inference speed of feature pyramid, the QueryDet^29^applies a query mechanism. In the first step of QueryDet, the coarse locations of small objects are predicted on low-resolution features. In the second step, the accurate detection results are computed on high-resolution features sparsely guided by those coarse positions. In the ES-Net^30^, five modules were designed to address the information loss of tiny objects and the mismatch between the receptive field of detection head and the scale of objects. These modules are the aggregated feature guidance module (AFGM), the split and aggregation enhancement module (SAE), the multi-receptive field fusion (MFF), the efficient stair pyramid (ESP), and the dynamic scale-aware head (DSH). In the SO-YOLO^31^, an expanding feature fusion of shallow features module was designed to effectively utilize the shallow features. Besides, the k-means++ clustering was applied to optimize the number and size of anchor box, and the redundant YOLO head network branches were removed.

### Attention mechanism

In general, the human visual system can quickly identify useful information from the external environment. Based on the above idea, various attention mechanisms^32^ are designed. The non-local neural networks^33^ is one of the most important works on attention mechanism. The non-local operations calculate the response at a position. Then, a weighted sum of the features at all positions is calculated and the remote dependencies are established through self-attention. This module can be applied to various tasks to improve the accuracy of models. The squeeze-and-excitation network (SENet)^34^, which focuses on the channel-level dependencies of the models is designed. In the SENet, the characteristic response value of each channel can be adjusted adaptively. Moreover, the convolutional block attention module (CBAM)^35^ is proposed. The CBAM combines channel attention and spatial attention to pay more attention to foreground objects. The selective kernel network (SKNet) with a selection kernel unit is proposed to adaptively adjust the size of the receptive field based on the input information.

The spatial and channel squeeze-and-excitation (scSE) is proposed for semantic segmentation. Besides, channel squeeze-and-excitation (cSE) and spatial squeeze-and-excitation (sSE) are designed simultaneously. Based on the combination of non-local neural networks and SENet, the global context network (GCNet) which utilizes a small number of calculations to optimize the global context modeling capabilities, is proposed. The criss-cross network (CCNet)^36^ is proposed based on non-local Neural Networks. The criss-cross attention module can be applied to capture contextual information from remote dependencies in a more effective way. The dual attention network (DANet)^37^ was proposed with two attention modules to a dilated fully convolutional network to model semantic dependencies in the spatial and the channel dimensions. The experimental results demonstrate that the proposed models achieve excellent performance on the Cityscapes dataset.

As above, there are various multi-scale feature fusion and attention mechanisms in the previous literature. However, most of the previous feature fusion mechanisms assumed that the weight of multi-scale feature maps was the same. Due to the small objects usually only appear in a part of the feature maps, it is helpful to detect small objects by assigning specific weight to each feature map. Therefore, this paper proposed a weight-based feature fusion module. Besides, a pyramids aggregation block is also designed to transform the pyramids into final pyramid.

## Proposed method

### The SSD benchmark model

In the baseline SSD, a feature pyramid is provided to detect multi-scale objects. To be specific, two layers $conv4\_3$ and *conv*7 of the backbone network and four newly added layers $conv8\_2$, $conv9\_2$, $conv10\_2$, and $conv11\_2$ are employed to construct the features pyramid. Therefore, six feature maps with the size of $38\times 38$, $19\times 19$, $10\times 10$, $5\times 5$, $3\times 3$, and $1\times 1$ are obtained. Each feature map is responsible for detecting specific scale objects. Based on the feature pyramid, SSD achieves excellent performance for detecting multi-scale objects. Let $P=\{p_1, p_2, p_3, p_4, p_5,p_6\}$ denote the feature maps, where $p_1$ represents the feature map with the largest resolution and $p_2$ means the feature map with the smallest resolution. Therefore, feature map $p_6$ captures more global information for detecting large-scale objects. However, the information on small-scale objects is gradually discarded from shallow to deep layers. Then, it is desirable to effectively the multi-scale feature maps to enhance the detection accuracy of SSD.

### WFFA-SSD architecture

**Figure 1:**
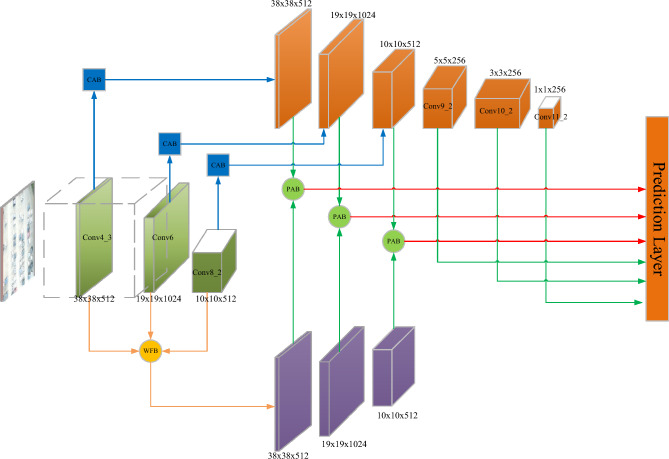
The structure of WFFA-SSD network.

To address the problem of scale variations in SSD, this paper proposes the weighted-based feature fusion and attention-based single-shot detector (WFFA-SSD). In the proposed method, two branches are constructed to generate two feature pyramids. The first one employs the context attention block to obtain an enhanced features pyramid. Another branch combines several feature maps with multiple scales based on the self-adaptive feature fusion block. Then, the second branch can also generate a feature pyramid. Next, two feature pyramids are merged to construct a new features pyramid to make a prediction. The architecture of WFFA-SSD is depicted in Fig. 1. The details of the proposed WFFA-SSD are described in the following subsections.

### Context attention block

The shape and texture of small objects are ambiguous in most images. However, there are some contextual relationships between the small-scale object and the other objects. Therefore, it is desirable to focus on the surrounding region and employ the context information for model representation. Therefore, this paper designs a context attention block (CAB) to capture the local scale context features. The encoding of the proposed CAB is implemented using the following operations Suppose that the output of any layer in the network is a feature map *F* and $F \in \mathbb (R)^{C\times H\times W}$, where *C* means the number of channels in *F*, *H*, and *W* denotes the height and width of *F*, respectively. *F* can be used as the input of CAB. At first, a convolution operator with a $3\times 3$ kernel attached by a dilation with $r=2$ is employed to acquire the larger receptive field. Considering the dilate convolution operator will reduce the continuity of the context information, a $1\times 1$ convolution is employed to smooth the information of features. To avoid over-fitting and accelerate the convergence of the training model, both convolution operators are followed by the ReLU activation function and batch normalization.

1 $$\begin{aligned} L(F)=BN(\sigma (Conv_2(BN(\sigma (Conv_1(F)))))) \end{aligned}$$

Moreover, the softmax function is employed to normalize the feature map.

2 $$\begin{aligned} G(F)=\frac{\exp {X_{i,j,k}}}{\sum _{i=1}\sum _{j=1}\sum _{k=1}\exp {X_{i,j,k}}} \end{aligned}$$

Then, the element-wise multiplication operator is applied to generate the new feature map.

3 $$\begin{aligned} F^{'}=G(L(F))\otimes F \end{aligned}$$

**Figure 2:**
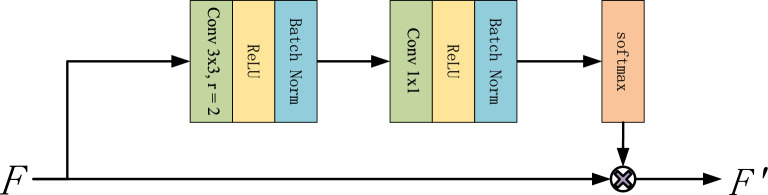
The structure of context attention block.

The output of CAB is $F^{'}$. The diagram of the CAB structure is shown in Fig. 2.

### Weight-based feature fusion block

**Figure 3:**
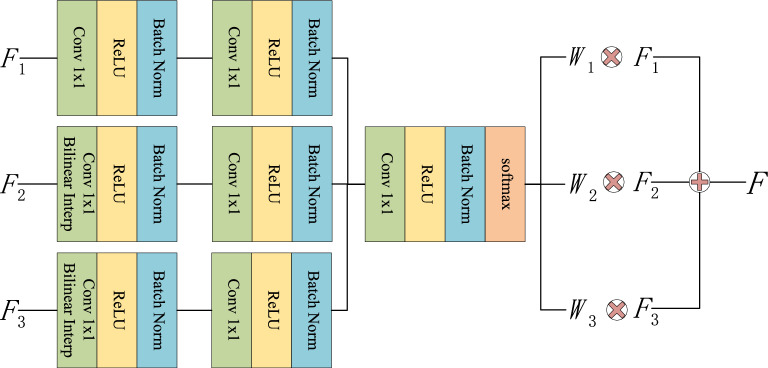
The structure of weight-based feature fusion block.

For a feature pyramid, the information in the same spatial location of multi-scale feature maps may be inconsistent. Therefore, the feature fusion block, which directly uses the element-wise addition or concatenation, cannot effectively take advantage of the feature maps of multiple pyramids. Based on the idea of ASFF^27^, this paper designs a weight-based feature fusion block (WFB) to make full use of the information of several feature maps to improve detection accuracy. The structure of WFB is depicted in Fig. 3.

Suppose three feature maps $F_1\in {\mathbb {R}}^{C_1\times H_1\times W_1}$, $F_2\in {\mathbb {R}}^{C_2\times H_2\times W_2}$, and $F_3\in {\mathbb {R}}^{C_3\times H_3\times W_3}$ are the input of WFB. As the resolutions as well as the channels of $F_1$, $F_2$, and $F_3$ are different, the $1\times 1$ convolution operator and the up-sampling operator are employed to obtain three feature maps $F_1^{'}$, $F_2^{'}$, and $F_3^{'}$ with the same scales. Then, the $1\times 1$ convolution operators are used to extract the weight of each position in each feature map. After that, the weight feature maps are concatenated along the channel dimension and the $1\times 1$ convolution operator is introduced to reduce the channel dimension to 3. Then, three weight feature maps $W_1$, $W_2$, $W_3$ can be obtained based on the softmax function. Finally, the fused feature map *F* can be obtained as follows.

4 $$\begin{aligned} F=W_1\times F_1 +W_2\times F_2 +W_3\times F_3 \end{aligned}$$

In this paper, the WFB is only used to obtain the fused feature maps based on the $38\times 38$, $19\times 19$, and $10\times 10$ feature maps. Then, the fused feature map is employed to generate a new feature pyramid.

### Pyramids aggregation block

**Figure 4:**
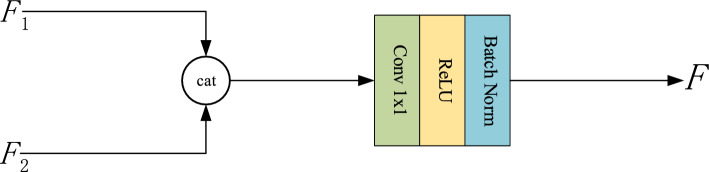
The structure of pyramids aggregation block.

In the proposed WFFA-SSD, two feature pyramids are generated based on the CAB and WFB. Then, a pyramids aggregation block (PAB) is devised to transform the pyramids into final detection pyramid. This module is illustrated in the Fig. 4.

The PAB consists of two steps. First, the same scale features in different pyramids are concatenated along the channel dimension. Then, the $1\times 1$ convolution operator with ReLU activation function and batch normalization is employed to smooth the feature maps and reduce the dimension.

## Experimental results and analysis

### Experimental setup

#### Dataset description

In this paper, the CARPK dataset is employed to experiment. The CAPRK dataset mainly focuses on car detection. From the perspective of unmanned aerial vehicles, the car is a small object. There are nearly 90,000 cars captured from four parking lots in CARPK. The resolution of the image is 1280 px $\times$ 720 px. The set of training and validation includes 989 images. Moreover, the test dataset contains 459 images. The CARPK dataset is available at https://lafi.github.io/LPN/.

PASCAL VOC dataset is a popular dataset for object detection. The PASCAL VOC consists of VOC2007 and VOC 2012. To be specific, there are 20 categories: airplane, bicycle, bird, boat, bottle, bus, car, cat, chair, cow, dining table, dog, horse, motorbike, person, potted plant, sheep, sofa, train, and TV monitor in PASCAL VOC. In this paper, 22,136 images of VOC2007 and VOC2012 are employed for training and validation. Then, 4952 images of VOC2007 are used for testing. The PASCAL VOC dataset is available at http://host.robots.ox.ac.uk/pascal/VOC/.

#### Evaluation metrics

In this paper, the mean average precision (mAP) is used to measure the detection accuracy, which is an indicator related to the IoU threshold. In this experiment, the most popular threshold IoU$=0.5$ is applied. In the multi-class objection detection problem, the mAP is the average value of the APs of multiple categories. Furthermore, the AP computes the area under the precision-recall curve. Therefore, the calculation procedure of precision and recall are given as follows.

5 $$\begin{aligned} Precision= & {} \frac{TP}{TP+FP} \end{aligned}$$

6 $$\begin{aligned} Recall= & {} \frac{TP}{TP+FN} \end{aligned}$$

where *TP*, *FP*, and *FN* represent the true positives, false positives, and false negatives, respectively. Furthermore, the frames per second (FPS) is adopted as the criterion of detection speed, which means the number of images the detection model can process per second with the specified hardware. In this paper, the FPS of each detector is tested on a single GPU device.

#### Implementation details

The experimental platform is a PC with 3.80GHz, 32GB RAM, windows 10 operating system. Our method is implemented by Python 3.7 based on the Pytorch framework, accelerating by a NVIDIA Geforce RTX 3080Ti GPU device with 12GB GPU memory, CUDA 11.4, and cuDNN 7.4.

In this experiment, the data augmentation of SSD is also done, and the related parameters are equal to SSD. Furthermore, the input images are resized to 300$\times$300$\times$3. In the training procedure, the detector is pre-trained on the combination of MS COCO, PASCAL VOC 2007, and PASCAL VOC 2012 datasets. Then the detector is fine-tuned on the CARPK dataset. The batch size is set to 32, the number of epochs is set to 300, and the initial learning rate is set to 1e-5 and decreased by 50% when the loss is not reduced for three consecutive epochs. The Adam algorithm is employed to optimize the loss function. The training objective is to minimize the weighted sum of Smooth L1 loss function $L_{loc}$ for localization and Softmax loss $L_{conf}$ for classification confidence as follows.

7 $$\begin{aligned} L(x,c,p,g)=\frac{1}{N}(L_{conf}(x,c)+\alpha L_{loc}(x,p,g)) \end{aligned}$$

where *N* represents the number of matched default boxes for random object *x*, *x* means the class confidence score, *p* indicates the predicted bounding box, and *g* represents the ground-truth bounding box. The hyper-parameter $\alpha$ is set to 1 by cross-validation.

### Ablation analysis

To verify the effectiveness of the blocks of WFFA-SSD, we construct two variants of WFFA-SSD and test the performance of variants on the CARPK dataset. The variants are the SSD-CAB and SSD-WFB. The results are reported in Table 1. First, the effect of the context attention block is tested. It can be observed the mAP of SSD-CAB is 84.93%, which is 2.21% higher than the SSD model. To further test the effect of the weight-based feature fusion block, the SSD-WFB is constructed based on SSD and WFB. The SSD-WFB obtains 85.82% mAP, which is 3.1% higher than SSD. When comparing the WFFA-SSD with the variants, the 86.96% mAP of WFFA-SSD is higher than the other variants. Therefore, it can be concluded that each block in WFFA-SSD is effective for the detection performance.

**Table 1:** Comparison of the proposed WFFA-SSD with the variants on CARPK dataset.

Method	Data	Backbone network	Speed (FPS)	Proposals	Input size	mAP (%)
SSD^9^	07+12	VGGNet-16	35.25	8732	300 × 300	82.72
SSD-CAB	07+12	VGGNet-16	29.00	8732	300 × 300	84.93
SSD-WFB	07+12	VGGNet-16	29.65	8732	300 × 300	85.82
WFFA-SSD	07+12	VGGNet-16	**28.41**	8732	300 × 300	**86.96**

Significant values are in [bold].

### Comparison with the state-of-the-art approaches on PASCAL VOC 2007 benchmark

In this experiment, the proposed WFFA-SSD model is compared with the baseline method and other state-of-the-art detectors on the PASCAL VOC 2007 benchmark. Since the proposed method is mainly modified based on SSD300^9^, SSD300 is chosen as the baseline method in the following experiments. The other object detectors including the two-stage detectors Faster R-CNN^8^ and R-FCN^38^, one-stage detectors SSD300^9^, SSD512^9^, DSSD513^39^, FSSD512^19^, YOLOv3^12^, and YOLOv4^40^. The results of the baselines except for the YOLOv4 are reported in the literature^41^. The results of YOLOv4 are obtained by running the code provided by the original literature on our machine.

**Table 2:** Comparison of the proposed WFFA-SSD with state-of-the-art detectors on the PASCAL VOC 2007 Test.

Method	Data	Backbone network	Speed (FPS)	Proposals	Input size	mAP (%)
Faster R-CNN	07+12	VGGNet-16	7	6000	600 × 1000	73.20
R-FCN	07+12	ResNet-101	5.8	300	600 × 1000	79.50
SSD300	07+12	VGGNet-16	46	8732	300 × 300	77.20
SSD512	07+12	VGGNet-16	19	24,564	512 × 512	78.50
DSSD513	07+12	ResNet-101	5.5	43,688	321 × 321	81.50
FSSD512	07+12	VGGNet-16	23.74	24,564	512 × 512	80.90
YOLOv3	07+12	DarkNet-53	46.94	10,647	416 × 416	79.61
YOLOv4	07+12	CSPDarkNet-53	**51.28**	10,647	416 × 416	87.2
WFFA-SSD	07+12	VGGNet-16	17.12	8732	300 × 300	**88.89**

Significant values are in [bold].

From Table 2, it can be observed that the proposed WFFA-SSD model achieves the highest mAP with 88.89% among 9 models. Compared with the series of SSD, the mAP obtained by WFFA-SSD is 7.39% higher than DSSD513 and 11.69% higher than the SSD300 baseline. Compared with the series of YOLO, the mAP obtained by WFFA-SSD is 9.28% higher than YOLOv3 and 1.69% higher than YOLOv4. Meanwhile, the detection speed of the detectors is also reported in Table 2. Compared with the series of R-CNN, the detection speed of WFFA-SSD is faster than Faster R-CNN and R-FCN. Besides, the detection speed of WFFA-SSD is also faster than DSSD513. However, the detection speed of WFFA-SSD is much slower than YOLOv3 and YOLOv4.

**Figure 5:**
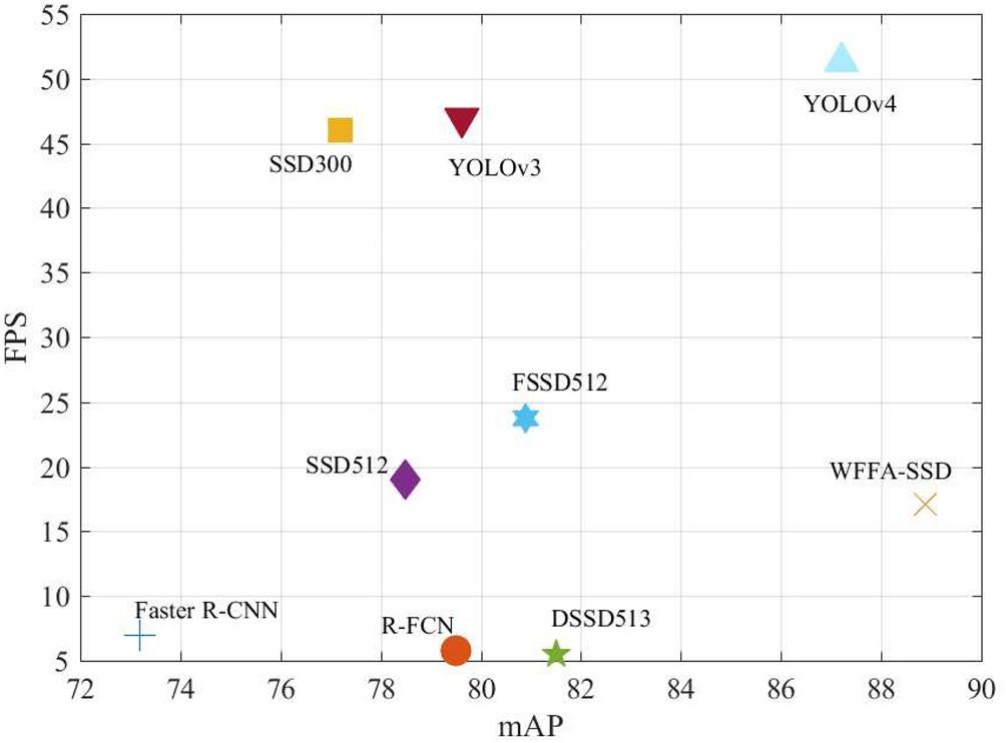
Speed and accuracy comparison of the proposed method with the state-of-the-art methods on the VOC dataset.

**Figure 6:**
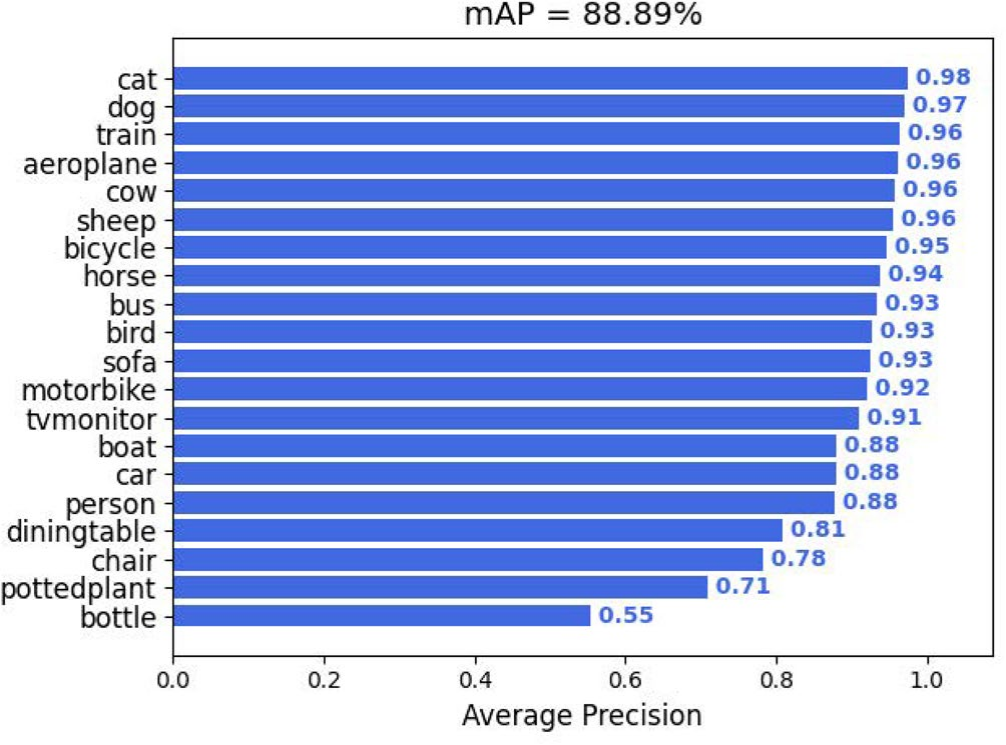
mAP of each object in PASCAL VOC dataset.

Figure 5 shows the speed and accuracy comparison of all models. It can be observed that the detection accuracy is far from satisfactory although the detection speed is slower than most of the one-stage detectors. Although the detection speed is slower than most one-stage detectors. The results cited here are reported by the literature^41^. From Fig. 6, it can be observed that the proposed WFFA-SSD achieves more than 90% mAP on 13 kinds of objects, and obtains lower than 70% mAP on bottles.

### Comparison with the state-of-the-art approaches on CARPK dataset

In this experiment, the effectiveness of the proposed WFFA-SSD is verified on the CARPK dataset. The WFFA-SSD is trained based on the pre-trained SSD model on the PASCAL VOC 2007, PASCAL VOC 2012, and MS COCO datasets. The compared baselines are Faster R-CNN, R-FCN, SSD300, YOLOv3, and YOLOv4. From Table 3, it can be observed that the proposed WFFA-SSD achieves the highest 86.96% mAP. To be specific, the mAP of proposed WFFA-SSD is 0.39% higher than YOLOv4, and is 0.95% higher than YOLOv3. When Compared with the two-stage detectors, the mAP of WFFA-SSD is 2.16% higher than Faster R-CNN, and is 0.83% higher than R-FCN. Meanwhile, the mAP of WFFA-SSD is 4.24% higher than the baseline SSD300. Furthermore, the detection speed of WFFA-SSD is 28.41 FPS, which is a tightly shower than 35.25% of SSD300. However, the detection speed of WFFA-SSD is also acceptable for real-time detection. The results of the baselines except for YOLOv4 are reported in the literature^41^.

**Table 3:** Comparison of the proposed WFFA-SSD with state-of-the-art detectors on the CARPK Test.

Method	Data	Backbone network	Speed (FPS)	Proposals	Input size	mAP (%)
Faster R-CNN	07+12+COCO	VGGNet-16	7.6	300	600 × 1000	84.80
R-FCN	07+12+COCO	ResNet-101	10.4	300	600 × 1000	86.13
SSD300	07+12+COCO	VGGNet-16	35.25	8732	300 × 300	82.72
YOLOv3	07+12+COCO	DarkNet-53	46.94	10,647	416 × 416	86.01
YOLOv4	07+12+COCO	CSPDarkNet-53	**50.42**	10,647	416 × 416	86.57
WFFA-SSD	07+12+COCO	VGGNet-16	28.41	8732	300 × 300	**86.96**

Significant values are in [bold].

**Figure 7:**
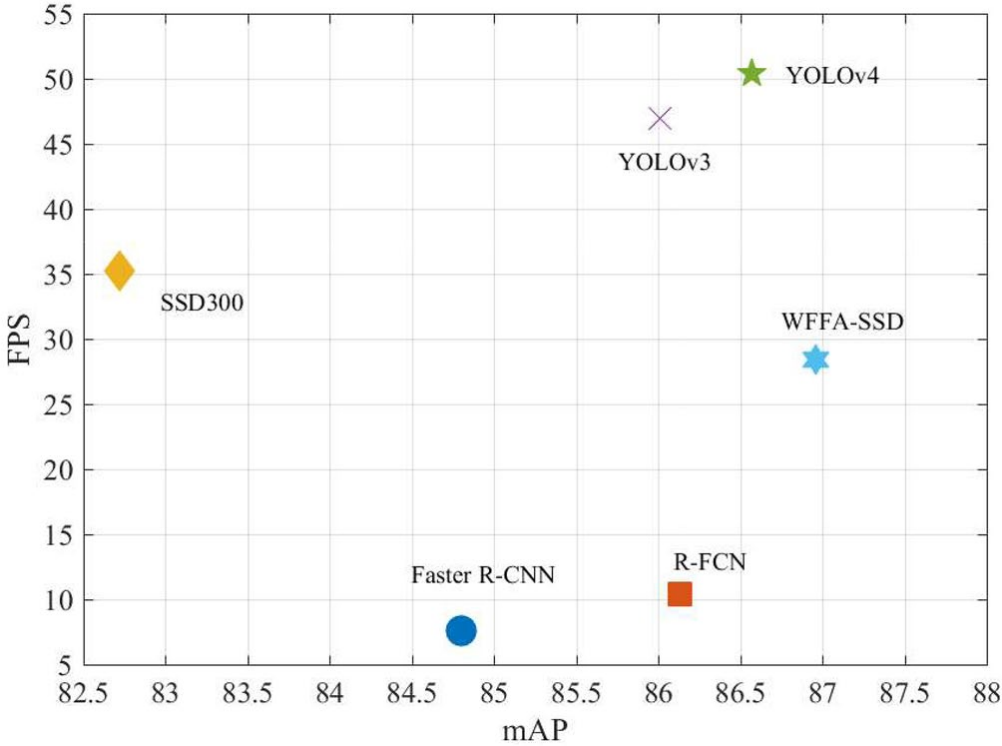
Speed and accuracy comparison of the proposed method with the state-of-the-art methods on the CARPK dataset.

The speed and accuracy comparison of WFFA-SSD with the compared models on the CARPK dataset is shown in Fig. 7. The WFFA-SSD can achieve a trade-off performance on the CARPK dataset.

**Figure 8:**
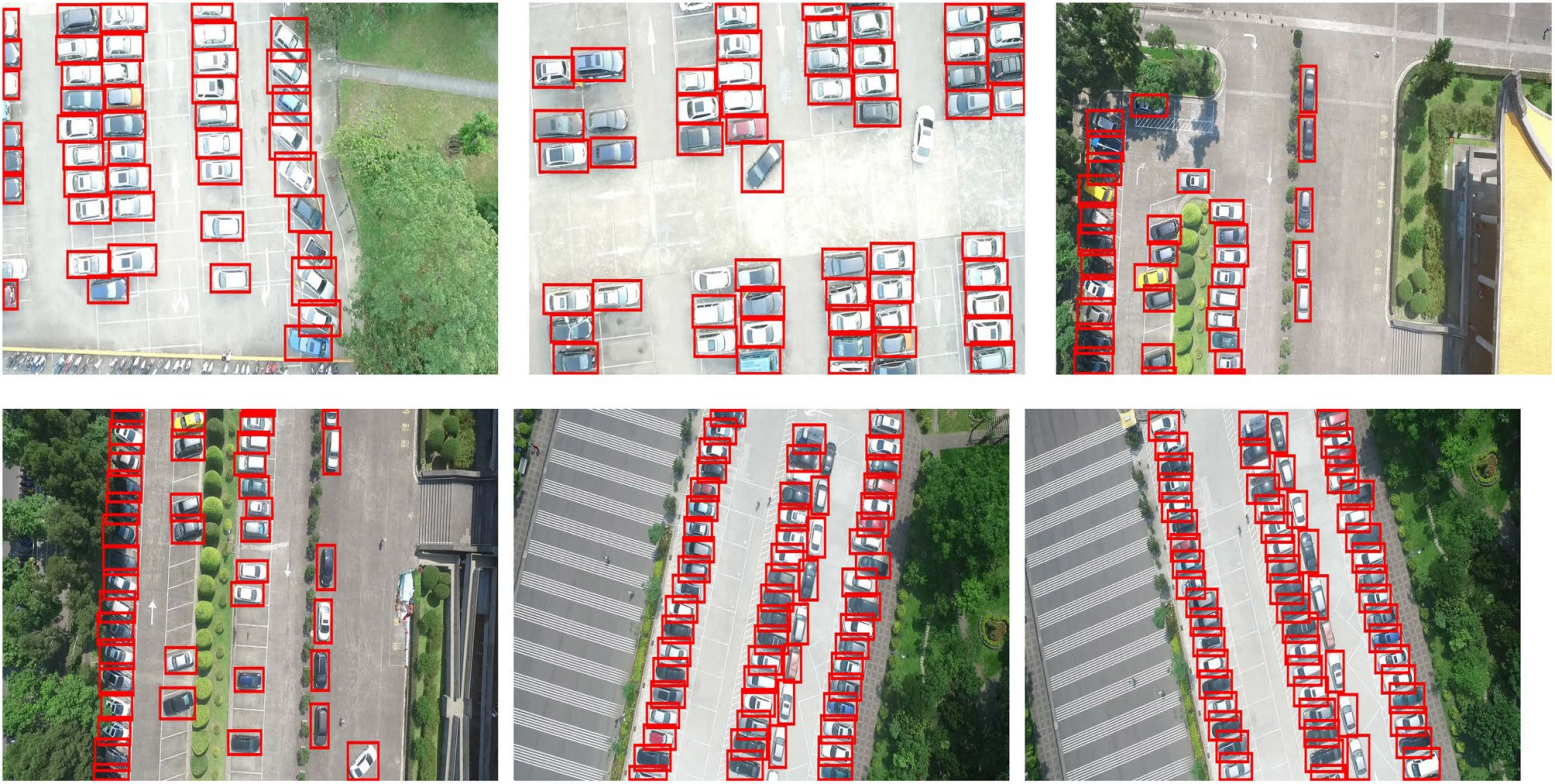
The detection results on CARPK dataset.

In this paper, three experiments are conducted to validate the effectiveness of the proposed WFFA-SSD. In the ablation analysis, the effectiveness of three blocks in WFFA-SSD has been analyzed. From Table 1, the complete WFFA-SSD outperforms the other variants. Each component of WFFA-SSD can enhance the accuracy of WFFA-SSD. In the comparison of WFFA-SSD with state-of-the-art baselines on the PASCAL VOC dataset, the WFFA-SSD achieves the highest mAP among the compared models. The reason can be summarized as follows. The weighted-based feature fusion can survive the useful information in different feature maps. Besides, the context attention block can enhance the accuracy of localization based on the large receptive field. In the comparison of WFFA-SSD with the other baselines on the CARPK dataset, the proposed WFFA-SSD outperforms the other compared models. The object detection results are shown in Fig. 8, which further indicates that the proposed WFFA-SSD is effective on the CARPK dataset.

## Conclusions and future work

This work is dedicated to developing a small objects detector. At present, the most effective strategy for the small object detection are feature fusion and attention mechanism. Therefore, the structures of the proposed detector and the previous detectors are similar. However, compared with most previous works, this work applies the weight-based feature fusion to adaptively adjust the weight of each feature map, and then multiple pyramids are aggregated into the final detection pyramid. According to the ablation analysis, it can be observed each block are an effort at the performance of WFFA-SSD. Moreover, the experimental results on the PASCAL VOC dataset demonstrate that the WFFA-SSD outperforms the compared models based on the novel blocks. Meanwhile, the results on the CARPK dataset indicate that the WFFA-SSD can effectively detect small objects on the CARPK dataset.

In future works, we will further improve the proposed blocks, and embed these blocks into more advanced baseline algorithms to achieve superior detection performance for small objects. In addition, it is necessary to explore real-time detection with a lightweight backbone network. Therefore, the detectors can be used in more scenes with limited computational resources. Besides, better training methods are also worth investigating.

## Data Availability

The datasets used and/or analysed during the current study available from the corresponding author on reasonable request.
